# Teaching and learning based on peer review: a realistic approach in forensic sciences

**DOI:** 10.12688/f1000research.8726.1

**Published:** 2016-05-31

**Authors:** Ricardo Jorge Dinis-Oliveira, Teresa Magalhães

**Affiliations:** 1 Department of Legal Medicine and Forensic Sciences, Faculty of Medicine, University of Porto, Porto, 4200-319, Portugal; 2 Department of Sciences, IINFACTS - Institute of Research and Advanced Training in Health Sciences and Technologies, University Institute of Health Sciences (IUCS), CESPU, CRL, Gandra, 4585-116, Portugal; 3 Department of Biological Sciences, UCIBIO-REQUIMTE, Laboratory of Toxicology, Faculty of Pharmacy, University of Porto, Porto, 4050-313, Portugal

**Keywords:** peer-review, teaching, learning, forensic sciences

## Abstract

Teaching and learning methods need a continuous upgrade in higher education. However it is also true that some of the modern methodologies do not reduce or prevent school failure. Perhaps the real limitation is the inability to identify the true reasons that may explain it or ignore/undervalue the problem. In our opinion, one of the current constraints of the teaching/learning process is the excess of and inadequate bibliography recommended by the teacher, which results in continuous student difficulties and waste of time in searching and selecting useful information. The need to change the paradigm of the teaching/learning process comes also from employers. They claim forensic experts armed with useful knowledge to face professional life. It is therefore mandatory to identify the new needs and opportunities regarding pedagogical methodologies. This article reflects on the recent importance of peer review in teaching/learning forensic sciences based on the last 10 years of pedagogical experience inseparably from the scientific activity.

## Introduction

Teaching and communicating with students is a very personal experience, shaped by the idiosyncrasy of each teacher but also dependent on the individual and motivational characteristics of the students as well as the institutional environment and the methodologies and pedagogical tools used by the teacher. Challenges of today’s society, particularly of economic, social and scientific nature, and the mass access to continuous new information, urges a revolution in the way of thinking on education. It is needed to renew the paradigm of teaching and it should be made every effort to ensure that the university and the professional life are seen as partners in research and education ^1^.

The model of teaching/learning based on the Bologna Process ^2^ designed to ensure comparability in the standards and quality of higher education qualifications, emphasizes the development of skills such as critical thinking, and attributes to the student an active role in his/her own training. It is obvious that these components are desirable and worth exploring, but there is still a long way to go. Resulting perhaps of a misunderstanding, the Bologna Process was sometimes assumed as an educational unaccountability of the teacher’s functions, transposing to the student almost the entire workload of the learning process. The challenge for higher education claims a continuous and systematic teaching/learning in order to build up capacities to define and solve problems, search for and select relevant scientific information, formulate hypotheses in an interdisciplinary basis, clearly communicate the results and draw conclusions. Indeed, learning is an ongoing process that occurs throughout life and is not limited and does not end in the syllabus of any curricular unit (academic discipline). Indeed, “once a student, always a student”. It is not only crucial, but also urgent aiming at reaching higher academic success rates that teachers assure detailed curricular plans, well-adjusted to the objectives, avoiding the huge amount of student’s workload almost impossible to accomplish. This is obvious regarding the frequent oversized bibliography recommendation.

In a system that favors the qualities developed and displayed in research, teaching is a field left to free will of each teacher and without proper investment and scrutiny by peers. It is for many teachers the poor relation of their work and the most often overlooked part. If the scientific component is subject to peer review, why do not take this reality also in teaching/learning process?

The purpose of this article is to reflect on the importance of the scientific peer review in teaching/learning forensic sciences and its inherent potential in offering a more reasonable and realistic education. This reflection is based on our teaching experiences and on our student’s feedback.

## Teaching methodologies

Since the last half of the twentieth century, most of the teaching methodologies, especially those applied in theoretical classes, were based on the transmission of information through transparency acetates, followed by slides and nowadays supported by datashow presentations. Teachers commonly assume that this type of documentation is the recommended study material, though not always prepared with the best quality and amount of information. Additionally, a non-criterions bibliography is recommended suggesting an “unaccountability” of the teacher of his/her functions. Indeed, is this bibliography of adequate size (often based on multiple sources not easily accessible to students) to the real available time for study/personal work? Do students effectively consult it, or on the other hand stay limited to the inefficiency of datashows presentations? Does the teacher have the right to request/recommend hundred pages after a single contact hour? Were the teachers able to achieve this transmission level in a packed auditorium with the continuous increase of the *numerus clausus*? Of course it is desirable and recommended that students develop routines for seeking information, but it is also important some dose of realism.

## Curricular syllabus and recommended bibliography

The development of the syllabus of any curricular unit is a privileged moment for reflection, particularly on the permanent adaptation of content and teaching methods to constant social changes of community life. It is also an excellent time for a look at our own journey as teachers in a permanent dissatisfaction behavior. Frequently, one of the great student difficulties is the immensity of bibliographic resources that can be used for study purposes. Although being excessive, recommended bibliography is insufficient to reverse the school failure. It is the teacher’s obligation to adjust and be realistic/reasonable when making available resources and contents. The exigence, rigor and seriousness of education is not measured by the number of pages that are recommended for study. At this level, the disrespect for those we teach transcends even the most skilled.

Nowadays, teaching Forensic Sciences acquires great importance in several academic areas such as medicine, pharmaceutical sciences, chemistry, biology, etc. ^3^. It refers broadly to the use of science in matters of law ^4^. It is used to identify a growing number of specialties and subspecialties, particularly in medicine and life sciences, that recurs to scientifically valid and legally admissible methods to clarify evidence that is being examined, or may be in the future, at judicial and judiciary level ^5^, under criminal or some other branch of the law. There are therefore separate disciplines within the Forensic Sciences ^6^. Noteworthy is that due to the multidisciplinary nature of forensic work and the way by which these sciences are organized in each country, it is not always easy to have useful bibliography for teaching Forensic Sciences. It was exactly this literature gap that prompted adjustments in the teaching/learning process of Forensic Sciences, leading to introducing peer review in classes preparation.

## Peer review in higher education

In the case of scientific literature, the peer review is an assessment of the soundness of the theme, originality and interest to the scientific community as well as the adequacy and accuracy of the methodology, results, discussion and conclusions, and also the relevance of citations. Although generally it does not ensure the veracity of content, peer review undoubtedly increases the quality of most scientific articles ^7, 8^.

Based on the importance that this peer has acquired in recent years, we have been particularly attentive and committed to use scientific scrutiny as a pedagogical opportunity to teach Forensic Sciences in the 1 ^st^, 2 ^nd^ and 3 ^rd^ Cycle of Studies, specialization and other continuing education courses that we coordinate ^3^. For this reason, several classes were written in a review/didactic article format, always aiming to adjust the recommended bibliography to what is actually taught in the classroom. These articles were then submitted for publication and are currently already available for students particularly in Forensic Toxicology. To accomplish this process it was crucial to respect the binomial education/research.

Unlike the requirements inherent to the most common datashow presentations, writing a scientific paper requires an additional effort in terms of time and reflection, which allows a good systematization of information and selection of literature. It produces consequently a non-comparable study document in terms of quality and usefulness. We believe that this educational pathway, besides being an effort of humility and scientific honesty aiming at the transmission of sound and useful knowledge, is also important to make students aware of the need of focusing their medical practice on scientific evidence and not just on empiric routines and mere opinions. Of course, writing classes in this format represents an immense team effort, but it is also rewarding. Although we have not, yet, a quantitative evaluation of academic success in pre- and post-publication scenario, much due to the fact that peer review is often a slow, sometimes expensive, subjective and prone to bias, there is no doubt that we have more satisfied students (including those already graduated and therefore more exigent), who effectively consult bibliography in the form of a scientific paper or book (and not limited to datashow presentations). It is also assured that students assimilate knowledge of useful quantity and quality and teachers are much more confident since the scrutiny of the peer review guaranties fewer errors and greater introspection regarding the topics focused in class. This type of teaching methodology represents also an effort of educating students aiming to transform the “campus in a laboratory” ^9^.

## Final remarks

Teaching and learning Forensic Sciences is based on a solid foundation of theoretical and/or practical knowledge promoting correct expert practices. It is important that the transmitted skills are based on proven scientific knowledge not only in autodidactic practices that leads over the years into vices and inevitable erroneous judicial decisions. Our practices for teaching and learning embodies some breaks with the past. One of them is the need for continuous evaluation of teachers by peer review, in order to improve students’ academic results.

As mentioned above, teaching and learning are ongoing processes and should promote the development of general and specific competences (in a particular professional field), and structure basic knowledge. In the age of entrepreneurship, realism is for us the philosophy of education. Therefore, it is legitimate to aim to a greater interconnection between academia and industry and offer students the knowledge of their practical life. To achieve this goal, it was truly useful and rewarding, to send classes in article format for peer review, in addition to the scrutiny carried out by the students through educational surveys. Pedagogical plurality and learning with peers are crucial aspects to better teach. In fact, this type of continuous teacher’s evaluation can represent an additional catalysis of learning ^10^. In full agreement with a recent *Nature* editorial ^11^, universities cannot continue with teaching methodologies based on the idea that only the most capable students will survive; we need to learn how to build a professor and researcher of the XXI century to have students with relevant competences. It is expected that peer review can be considered as a parameter for increasing the quality of classes in higher education.
