# Supplementary material for: Teaching and learning based on peer review: a realistic approach in forensic sciences
Source: F1000Res. 2016 May 31;5:1048. [Version 1] doi: 10.12688/f1000research.8726.1 (PMC4975368; doi:10.12688/f1000research.8726.1)
Supplement: Peer review history

# Peer review: Referee response for version 1

**DOI:** 10.5256/f1000research.9389.r15085

**Published:** 2016-08-04

**Authors:** Mauricio Yonamine, Flavia Lopes Roveri

**Affiliations:** 1 Department of Clinical and Toxicological Analysis, School of Pharmaceutical Sciences, University of São Paulo, São Paulo, Brazil; 2 University of São Paulo, São Paulo, Brazil

Forensic science needs a multidisciplinary approach and requires knowledge from different areas such as sciences of life and death, natural sciences, technology and law from students. In fact, its complexity demands new pedagogical methodologies, in view that conventional classes based on datashow presentations and complementary studies in multiple bibliography sources do not seem to be effective in the learning process. Based on this context, the discussion raised by the authors is completely valid and their arguments seem to be logical. Certainly this teaching strategy is much more laborious, however, it can generate contemporary knowledge for both professors and students, providing the opportunity to receive other expert´s opinions on the covered topic. We believe that the authors could enrich the discussion if more results about the teaching methodology were presented, including the student’s opinion, which would enhance the manuscript and strengthen this practice. In addition, the process of teaching and learning could also be improved with the presence of professionals in the area, as forensic experts. Their experience acquired in cases resolution could be taken to the classroom, which would be an opportunity for students to apply the acquired theoretical knowledge in real forensic cases. Students could discuss about the proposed problem, presenting their hypothesis and revealing their understanding. In the end, the case resolution could also be presented in the form of a forensic case report and submitted to the peer review process. By doing so, the subject would present theoretical and practical strenght.

We have read this submission. We believe that we have an appropriate level of expertise to confirm that it is of an acceptable scientific standard.

# Peer review: Referee response for version 1

**DOI:** 10.5256/f1000research.9389.r14499

**Published:** 2016-06-21

**Authors:** Hugo Filipe Violante Cardoso

**Affiliations:** 1 Department of Archaeology, Centre for Forensic Research, Simon Fraser University, Burnaby, BC, Canada

The reflection provided in this article is scientifically solid and sound. The abstract summarizes well the contents of the published work and, although the title is appropriate, it carries some ambiguity about what exactly the review process refers to. This would probably require a rather long and inconvenient title and, therefore, the current one seems appropriate. The paper is of a reflective nature, but outlines clearly the problem, the particular solution that was undertaken in an attempt to solve it, and some of the results. It would have been interesting to read more details about the results of the peer-review process itself and the actual impact in the classroom, but this is perhaps a first attempt at materializing some of the authors’ thoughts and experience that will be developed further in other publications or reports. The conclusion consolidates the authors’ opinion that peer-review as a pedagogical tool enhances the connection between scientifically generated knowledge and forensic practice in a higher education setting. As an article that focuses on the teachers’ wishes to improve on lecture delivery quality and teachers’ pedagogic accountability, it successfully outlines a classroom approach that has much to offer. I am certain that many teachers will be looking forward to knowing more about the learning outcomes of this approach and will give it strong consideration as a pedagogical tool.

I have read this submission. I believe that I have an appropriate level of expertise to confirm that it is of an acceptable scientific standard.
